# The relationship between negative life events and quality of life in adolescents: Mediated by resilience and social support

**DOI:** 10.3389/fpubh.2022.980104

**Published:** 2022-09-23

**Authors:** Yinshuang Tang, Yingjie Ma, Jinglin Zhang, Hong Wang

**Affiliations:** ^1^Department of Maternal and Child Health and Adolescent Health, School of Public Health, Chongqing Medical University, Chongqing, China; ^2^Department of Epidemiology and Health Statistics, School of Public Health, Chongqing Medical University, Chongqing, China

**Keywords:** negative life events, resilience, social support, quality of life, adolescents

## Abstract

**Background:**

Quality of life has become an important indicator for assessing the health care of adolescents. This study aimed to explore the relationship between negative life events and quality of life in adolescents and the potential mediating roles of resilience and social support.

**Methods:**

A stratified cluster sampling technique was used to select 3,860 adolescents as study participants. The Adolescent Self-Rating Life Events Checklist, the Resilience Scale for Chinese Adolescents, the Social Support Rating Scale, and the Adolescent Quality of Life Scale were used by participants to rate their negative life events, resilience, social support, quality of life, respectively. The correlations between study variables were analyzed by the Pearson correlation analyses. The AMOS 26.0 software was used to explore the mediating roles of resilience and social support in negative life events and quality of life.

**Results:**

There was a negative correlation between negative life events and quality of life (β=-0.745, *P* < 0.05); resilience and social support played an important mediating role in the relationship between negative life events and quality of life (β_Resilience_ = −0.287, *P* < 0.05; β_Social support_ = −0.124, *P* < 0.05). The emotional adjustment dimension of resilience (β = −0.285, *P* < 0.05) and the subjective support dimension of social support (β = −0.100, *P* < 0.05) played the largest mediating roles, respectively.

**Conclusion:**

Negative life events were negatively correlated with adolescents' quality of life. Strengthening resilience and social support is expected to weaken and reduce the adverse effects of negative life events on adolescents and further maintain and improve their quality of life.

## Introduction

The World Health Organization (WHO) defines quality of life (QoL) criteria as an individual's perception of their place in life in the context of the culture and value system in which they live and their goals, expectations, standards, and concerns (1). It is generally considered to be a multidimensional structure and its evaluation usually depends on individuals' subjective evaluation of well-being and/or function in the different domains that make up the overall structure (2). Research on adolescents shows that QoL is about the positive cycles of life. To enter and maintain a positive cycle, a positive self-image, good friends, and strong family relationships are important. Consequently, adolescents' QoL is threatened when these factors are negative (3).

Adolescence is a critical period of transition from childhood to adulthood involving rapid physical, psychological, and social changes that shape adolescents' personality, behavior, and future health (2). Negative life events [NLEs; (4)], such as interpersonal strain and excessive pressure of study, are key risk factors for adolescent development and have a significant impact on the psychological and social outcomes of adolescents (5–7). Although personality characteristics play an important role in an individual's psychological and social development, environmental experience also influences these outcomes. Compared with adults, adolescents are more sensitive to the perception of the environment and more vulnerable to self-evaluation and external factors (8). The experience of NLEs in childhood can stimulate negative cognition and behaviors and become contributing factors to depression and other psychological problems in adulthood (9). The overall results of the current study suggest that NLEs are associated with increased psychological stress and reduced life satisfaction (10, 11). The occurrence of major NLEs directly predicts lower QoL (12, 13).

Resilience is defined as a personal quality that enables a person to thrive in the face of adversity. People with a higher level of resilience are typically characterized by optimism, positive coping, and hardiness, which may help them successfully cope with NLEs and maintain better mental and physical health outcomes (14). Previous studies have shown that NLEs impair resilience (15), and that resilience is a positive predictor of adolescents' QoL (16, 17). According to Kumpfer's mental resilience framework theory (18), people have different degrees of mental resilience due to different cognitive, emotional, and mental states. When feeling the pressures or challenges of the outside world, an individual's resilience will respond to the pressure from the outside world. High resilience individuals can adapt to these pressures but low resilience individuals fare less well. Resilience can also play an intermediary role (18). For example, resilience was found to be a mediator between NLEs and positive social adjustment (15), and between self-compassion and QoL (19). That is, resilience may act as a factor directly affecting health-related outcomes and also play a role as a mediator. Resilience is a multidimensional concept that has recently been identified as a potentially modifiable factor (20) that can be improved through intervention (21). It is our premise that resilience is the mediator between NLEs and QoL. Thus, when individuals experience NLEs, which are often unavoidable, resilience can help them maintain and promote their mental health and QoL (22).

Social support refers to external support from important others and society (23). According to the stress-buffering hypothesis, social support may help individuals redefine stressful situations so that they are no longer perceived as stress and/or supply resources, thereby reducing the severity of stressful events (24). The Convoy model of social relations (25) and Novena's conceptual model of meaning in life (26) further reveal the relationship between life satisfaction, living arrangements, social support, and meaning in life. The Convoy model suggests that social support from different social networks (such as living arrangements) plays an important role in determining individual subjective well-being and life satisfaction (25). According to Novena's conceptual model, conditions such as living arrangements can serve as a foundation for the components that give meaning to life. One study showed that social support plays an intermediary role between living arrangements and life satisfaction (27). Another study showed that social support mediates the relationship between NLEs and depression in adolescents (28). QoL is a response to life events and changes in the living environment; adolescents' social support is positively related to their QoL (29, 30). However, our understanding of the role of social support in NLEs and QoL is rather limited. Thus, our goal is to explore whether social support plays a mediating role in NLEs and QoL. If so, the introduction of social support for adolescents should improve their QoL.

**Figure 1:**
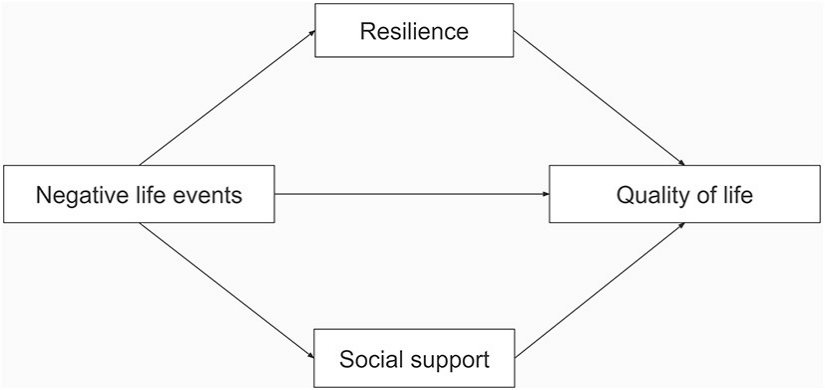
The hypothesized model.

Previous research has examined the relationship between NLEs and QoL, resilience and QoL, and social support and QoL. To the best of our knowledge, no study has investigated the mediating role of resilience and social support in NLEs and QoL, especially among adolescents. Therefore, in 2020, we conducted a population-based survey among primary and middle school students in Chongqing, China, to examine the relationship between NLEs and QoL in children and adolescents, and to explore the mediating role of resilience and social support. Based on previous studies, we hypothesized that (1) NLEs were negatively correlated with QoL in adolescents, (2) resilience and social support were negatively correlated with NLEs and positively correlated with QoL, and resilience and social support played a mediating role between NLEs and QoL. Our hypothesized model can be seen in Figure 1.

## Materials and methods

### Study design, participants, and process

The stratified cluster sampling method was used to select the study sample in Qijiang District, Chongqing. As per the stratification of urban and rural areas, two primary schools and two middle schools were randomly selected from urban and rural areas of the region, respectively. The study participants—primary school students from grades 4–6 and junior middle school students from grades 7 to 9—were selected from a total of eight schools. The formal survey was conducted in November 2020 with a sample size of 3,860 participants. The inclusion criterion was that students could understand and complete the questionnaire independently. The study was approved by the ethical committee of Chongqing Medical University and written informed consent was obtained from students and their parents before the research study began.

Participants were surveyed using a self-reported questionnaire, which included general demographic characteristics such as gender and grade, as well as scales related to NLEs, resilience, social support, and QoL. The questionnaire investigators were composed of two professors and six graduate students. Before the survey, the investigators were given standardized training by a professor to ensure that they had a clear understanding of the structure of the questionnaire and how to fill in the questionnaire. Under the supervision of the school leaders, the investigators entered each sample class in the school to conduct a field survey of the students. After they explained how the questionnaire should be filled out, the students completed the questionnaire in the classroom with the help of the investigators and the head teacher. Participants were assured that their responses would be confidential and anonymous. After the students completed the questionnaires, the investigators checked and collected the questionnaires one by one and timely returned the questionnaires with missing or logical errors to the participants for supplementary information and modification.

### Instruments

#### General questionnaire

According to the influencing factors of adolescents' QoL (31–33), the following sociodemographic characteristics were collected using the self-designed questionnaires: age (continuous variable); gender (male/female); grade (4–9); whether the individual is a left-behind child (yes/no); family economic status (low/moderate/high); academic stress (low/moderate/high). Left-behind children refer to those who remain at home and are cared for by other family members or caregivers because one or both parents have migrated to jobs outside of their home region (34). All questions were self-reported by the participants.

#### Negative life events

The Adolescent Self-Rating Life Events Checklist [ASLEC; 15] was used to assess the impact of NLEs on adolescents in the past 12 months. It consisted of 27 items that covered six dimensions—interpersonal relationships, learning pressure, punishment, bereavement and property loss, health and adaptation, and others. Each item was assessed with a 5-point Likert scale to rate the influence of NLEs on adolescents—no influence (1), mild influence (2), moderate influence (3), severe influence (4), and extremely severe influence (5). The higher the total score, the greater the impact of NLEs. The Cronbach's alpha of this measure was 0.889 (95% *CI* [0.884, 0.894]). The Cronbach's alpha coefficients of interpersonal relationships, learning pressure, punishment, bereavement and property loss, health and adaptation, and others were 0.735 (95% *CI* [0.721, 0.748]), 0.594 (95% *CI* [0.574, 0.614]), 0.678 (95% *CI* [0.663, 0.694]), 0.584 (95% *CI* [0.523, 0.572]), 0.414 (95% *CI* [0.383, 0.444]), and 0.581 (95% *CI* [0.559, 0.602]), respectively.

#### Quality of life

The Adolescent Quality of Life Scale (35) was used to measure QoL. The scale consisted of 39 items, divided into four dimensions, as follows: physical (8 items), psychological (11 items), pubertal (6 items), and social (14 items), which were used to measure the QoL of children and adolescents. Each item was scored on the 5-point Likert scale. If the item was related to the frequency of a certain phenomenon, it was scored from 5 to 1, that is, from “never” to “always”. If the item was satisfactory for a certain situation, it was rated from 1 to 5 points, that is, from “very dissatisfied” to “very satisfied.” The higher the score, the better the QoL. The overall Cronbach's alpha was 0.923 (95% *CI* [0.920, 0.927]). The Cronbach's alpha coefficients of physical, psychological, pubertal, and social were 0.869 (95% *CI* [0.863, 0.876]), 0.821 (95% *CI* [0.812, 0.829]), 0.878 (95% *CI* [0.872, 0.883]), and 0.606 (95% *CI* [0.586, 0.625]), respectively.

#### Resilience

The Resilience Scale for Chinese Adolescents (RSCA) (36) was used to measure resilience and included five factors, as follows: target focus, emotional adjustment, positive cognition, family support, and interpersonal assistance. This scale contained 27 items and was scored on a 5-point Likert scale. Positive items were rated on a scale of 1–5, from “completely inconsistent” to “completely consistent.” Negative items were rated in reverse. The higher the score, the higher the psychological resilience. In this study, the Cronbach's alpha value of the overall scale was 0.853 (95% *CI* [0.846, 0.859]). The Cronbach's alpha coefficients of target focus, emotional adjustment, positive cognition, family support, and interpersonal assistance were 0.794 (95% *CI* [0.784, 0.804]), 0.743 (95% *CI* [0.730, 0.755]), 0.747 (95% *CI* [0.733, 0.759]), 0.687 (95% *CI* [0.671, 0.702]), and 0.710 (95% *CI* [0.696, 0.724]), respectively.

#### Social support

The Social Support Rating Scale [SSRS; 37] was used to detect the degree of support received by participants in social life and their utilization of the support. This scale consisted of 10 items comprised of three dimensions, as follows: objective support, subjective support, and utilization of social support. Items 1–4 and 8–10 were scored on a four-point scale (1–4 points). Item 5 was divided into five sub-questions, and each sub-question was graded from “none” to “full support” on a 1–4 point scale. For items 6 and 7, points were given according to the number of sources in the “following sources.” If the answer was “no source,” the score was 0. The higher the total score, the higher the overall social support. The overall Cronbach's alpha was 0.717 (95% *CI* [0.704, 0.730]). The Cronbach's alpha coefficients of subjective support, objective support, and utilization of social support were 0.543 (95% *CI* [0.519, 0.566]), 0.497 (95% *CI* [0.468, 0.523]), and 0.600 (95% *CI* [0.578, 0.621]), respectively.

### Data analysis

SPSS 24.0 and AMOS 26.0 software were used to analyze the data. Descriptive analyses, including frequency and percentage or mean and standard deviation, were calculated for describing participants' sociodemographic characteristics. The Pearson correlation analysis was used for the correlation analysis of study variables—NLEs, resilience, social support, and QoL. The AMOS 26.0 software was used to explore the mediating roles of resilience and social support in NLEs and QoL. Due to the theoretical crossover between the dimensions of family support and interpersonal assistance in resilience and the three dimensions of social support, the mediating effect model of resilience and social support, directly constructed may lead to multicollinearity between resilience and social support. In this study, the influence of multicollinearity was eliminated by setting a constraint condition to connect the residual term of resilience and social support in the total dimensional analysis. In the dimensional analysis, the residual terms of the two dimensions of family support and interpersonal assistance in resilience and the three dimensions of social support were connected to eliminate the influence of multicollinearity. The 2,000 bias-corrected samples were extracted to calculate the path coefficients and their significance. A confidence interval (*CI*) without 0 indicated a significant mediating effect. A *P*-value (two-tailed) < 0.05 was considered statistically significant.

## Results

### Descriptive data and correlations

Descriptive statistics of the sociodemographic characteristics of the participants are shown in Table 1. The sample consisted of 1,925 (49.87%) male and 1,935 (50.13%) female students with an average age of 12.51 years. There were 1,756 (45.49%) primary school students and 2,104 (54.51%) junior high school students, including 1,579 (40.91%) left-behind students. Among them, 2,138 (55.39%) students assessed their family economic status as “moderate”, 1,297 (33.60%) students assessed their family economic status as “high,” and 425 (11.01%) students assessed themselves as “low”. The self-rated learning pressure of students was as follows: 2,027 (52.51%) students rated their learning pressure as “moderate”, 538 (13.94%) students as “low”, and 1,295 (33.55%) students as high.

**Table 1:** Sociodemographic characteristics of participants (*n* = 3,860).

**Characteristics**	**Mean or *n***	**SD or %**
Age (years)	12.51	1.66
**Gender**
Male	1,925	49.87
Female	1,935	50.13
**Educational degree**		
Primary school	1,756	45.49
Middle school	2,104	54.51
**Left behind children**
Yes	1,579	40.91
No	2,281	59.09
**Family economic status**
Low	425	11.01
Moderate	2,138	55.39
High	1,297	33.60
**Academic stress**
Low	538	13.94
Moderate	2,027	52.51
High	1,295	33.55

Quantitative data were expressed by “mean” and “standard deviation (SD)”, qualitative data were expressed by “n” and “percentage (%)”.

The correlation analysis of each variable is shown in Table 2. As previously assumed, all variables were significantly correlated. The NLEs were negatively correlated with resilience, social support, and QoL. Resilience was positively correlated with social support and QoL. Social support was positively correlated with QoL.

**Table 2:** Correlations, means, and standards deviations of study variables.

	**Mean**	**SD**	**NLEs**	***R***	**SS**	**QoL**
NLEs	40.71	11.25	1			
R	91.67	17.01	−0.480*	1		
SS	40.94	7.02	−0.393*	0.585*	1	
QoL	148.07	20.45	−0.636*	0.627*	0.527*	1

SD, standard deviation; NLEs, negative life events; R, resilience; SS, social support; QoL, quality of life.

^*^P < 0.05.

### Mediation analyses

**Figure 2:**
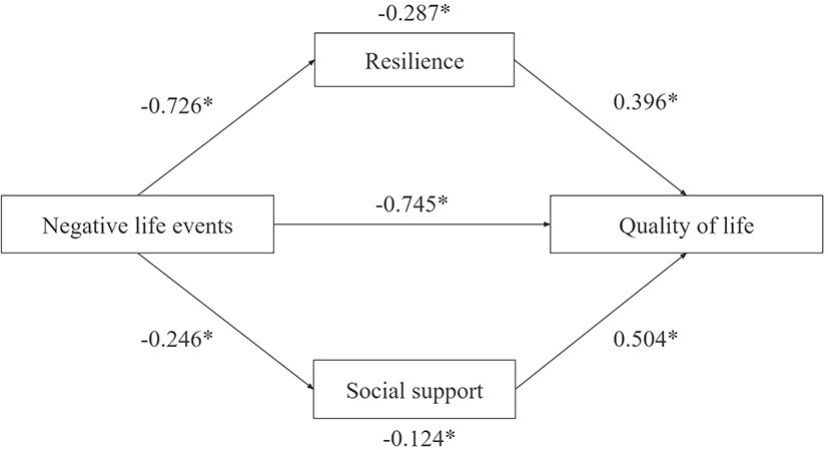
Mediation model. *P < 0.05.

As shown in Table 3, the mediating effect (indirect effect) of NLEs on QoL was significant (β = −0.411, 95% *CI* [−0.448, −0.381]). The direct effect of NLEs on QoL was also significant (β = −0.745, 95% *CI* [−0.805, −0.690]). Specifically, both resilience and social support mediated the relationship between NLEs and QoL. NLEs significantly predict decreased mental resilience (β = −0.726, 95% *CI* [−0.777, −0.674]), social support (β = −0.246, 95% *CI* [−0.267, −0.225]), and lower QoL (β = −0.745, 95% *CI* [−0.805, −0.690]). The mediating effect of resilience (β = −0.287, 95% *CI* [−0.322, −0.255]) and social support (β =-0.124, 95% *CI* [−0.149, −0.100]) accounted for 24.85% and 10.72% of the total effect, respectively (Figure 2).

**Table 3:** The mediating effect of NLEs on QoL through R and SS.

**Outcome**	**Mediation analysis paths**	**Estimated**	**95% bias-corrected** ***CI***	
			**Lower**	**Upper**
QoL	Total effect	−1.157	−1.219	−1.093
	Direct effect	−0.745	−0.805	−0.690
	Indirect effect	−0.411	−0.448	−0.381
	NLEs → R	−0.726	−0.777	−0.674
	R → QoL	0.396	0.356	0.433
	NLEs → SS	−0.246	−0.267	−0.225
	SS → QoL	0.504	0.413	0.598
	NLEs → QoL	−0.745	−0.805	−0.690

NLEs, negative life events; R, resilience; QoL, quality of life; SS, social support; CI, confidence interval.

Table 4 further demonstrates the mediating effects of various dimensions of resilience and social support on the relationship between NLEs and QoL. Among them, emotional adjustment in resilience had the greatest mediating effect on the relationship between NLEs and QoL (β = −0.285, 95% *CI* [−0.317, −0.257]). In social support, the subjective support dimension had the largest mediating effect (β = −0.100, 95% *CI* [−0.123, −0.079]).

**Table 4:** The mediating effect of NLEs on quality of life through various dimensions of R and SS.

**Outcome**	**Mediation analysis paths**	**Estimated**	**95% bias-corrected** ***CI***	
			**Lower**	**Upper**
QoL	NLEs → R_1_ → QoL	−0.040	−0.054	−0.029
	NLEs → R_2_ → QoL	−0.285	−0.317	−0.257
	NLEs → R_3_ → QoL	0.006	−0.002	−0.011
	NLEs → R_4_ → QoL	−0.049	−0.070	−0.029
	NLEs → R_5_ → QoL	0.016	0.035	0.002
	NLEs → SS_1_ → QoL	−0.100	−0.123	−0.079
	NLEs → SS_2_ → QoL	0.008	−0.001	0.016
	NLEs → SS_3_ → QoL	−0.054	−0.070	−0.039

NLEs, negative life events; R_1_, target focus; R_2_, emotional adjustment; R_3_, positive cognition; R_4_, family support; R_5_, interpersonal assistance; QoL, quality of life; SS_1_, objective support; SS_2_, subjective support; SS_3_, utilization of social support; CI, confidence interval.

## Discussion

The purpose of this study was to explore the influence of NLEs on adolescent QoL and its potential mediating mechanism. As hypothesized, our results show a significant negative association between NLEs and adolescent QoL, which is consistent with previous studies (10, 11). Importantly, we found that resilience mediated the relationship between NLEs and QoL in adolescents. The mediating effect value was −0.287, accounting for 24.85% of the total effects. Most people will inevitably be exposed to traumatic experiences during their lives, which have been shown to negatively impact physical and mental health (37–39).

Resilience is becoming an increasingly popular concept for research and application in the field of prevention (18). Adolescence is the developmental period with the highest risk for experiencing multiple types of NLEs, such as academic stress, injuries, interpersonal violence, and chronic diseases (40–42). Studies have shown that frequent exposure to NLEs in childhood and adolescence is associated with poorer, health-related QoL (43, 44). With reduced funding for services to help children and families at risk, there is an urgent need for information on low-cost ways to increase children's resilience to negative life events. A better understanding of ways to increase the resilience of all children holds great promise for improving the effectiveness of prevention services in communities, schools, and families (18). Resilience is considered an important aspect of health and well-being and refers to the ability to cope with difficulties, stress, and trauma while maintaining or restoring normal function. It is regarded as a complex dynamic process (45) that is influenced by psychological, social, and biological factors (46). The higher the resilience, the lower the vulnerability and risk of disease (47).

The results of this study showed that resilience was positively correlated with QoL (16), and negatively correlated with NLEs (17), which is consistent with the results of previous studies. Therefore, it is reasonable to infer that NLEs could reduce the level of resilience and further predict lower QoL. In addition, the results suggest that a targeted intervention for improving resilience can help individuals to resist the negative impact of NLEs on QoL and maintain and improve QoL. In this study, emotional control of resilience played the most important mediating role. Previous studies have found that the tendency to feel and share positive emotions, especially those related to pleasant events, has a strong interpersonal component that can improve QoL (48). In contrast, the negative emotions of sadness, anger, guilt, disgust, shame, and embarrassment affect the way stressors are assessed and can have a damaging effect on life satisfaction (49, 50). Thus, it is expected to reduce the negative impact of NLEs on QoL by improving the individual's ability to manage their emotions with respect to resilience.

Social support is described as “the support an individual receives through social networks with others” (51). In this study, we found that social support was not only significantly associated with NLEs and QoL but also mediated the effects on the relationship between NLEs and QoL in adolescents. The mediating effect value was −0.124 and accounted for 10.72% of the total effects. Adolescence is the main transitional period of an individual's life and is characterized by biological, behavioral, and psychological changes under the influence of social conditions and family characteristics (52). With the change and expansion of social relations among family members, teachers, and friends, the interdependence and influence between individuals and different social groups are increasing, and social support is closely related to the emotional and social development of adolescents (53). According to social support theory, the stronger a person's social support network, the better able they are to cope with environmental challenges. Social support may influence adolescents' assessment of situations, improve their problem-solving skills, and promote adaptive behavior (54). Previous studies have demonstrated that social support can buffer the impact of NLEs on depression, anxiety, and other psychological problems (24, 28, 55). Social support from the school, family, and other environments is a positive predictor of adolescents' QoL (29, 56).

In this study, social support, as a protective factor of QoL, is a mediator between NLEs and QoL and can buffer the negative impact of NLEs on QoL in adolescents. Among them, the subjective support dimension plays the strongest intermediary role. Subjective support is also called perceived social support, that is, the emotional support experienced by individuals, and the emotional experience and satisfaction of individuals who are respected, supported, and understood in society. Perceived social support is closely related to the subjective feelings of individuals (57). Crockett et al. (58) found that having a strong family social support network can enable individuals to have better emotional regulation and protect them from external stress. Social work with a social support theory orientation emphasizes the intervention of an individual's social network to change its role in the individual's life. Individuals with insufficient social support resources or insufficient ability to use social network need social workers to work with them to reduce the negative impact of NLEs on QoL. Specifically, social workers can help these individuals to expand their social support resources, enhance their emotional support, and improve their ability to use social networking networks.

Adolescence is a time of many changes characterized by an increasing trend in exposure to NLEs (59), and a gradual decline in life satisfaction in terms of cognitive evaluation of overall life (60, 61). In 2015, the H6 agencies (United Nations Program on HIV/AIDS, United Nations Population Fund, United Nations Children's Fund, UN Women, WHO, and the World Bank Group) launched the Global Strategy for Women's, Children's and Adolescent's Health (2016–2030), which identifies adolescents as central to achieving the United Nation's Sustainable Development Goals [SDGs; (62)]. In 2019, China issued the Healthy China Action plan (2019–2030), which aims to promote the health and well-being of children and adolescents through a series of steps and programs (63). These policies emphasize the importance of adolescents' QoL. To better promote teenagers' QoL, it is urgent to find and use effective intervention approaches. Given that the NLEs experienced by adolescents are not always avoidable, traditional education alone may not be effective. Based on the results of this study, targeted interventions on resilience and social support may reduce the impact of NLEs on adolescents so they can enjoy a better QoL.

## Limitations of the study

This study has some limitations that need to be considered. Firstly, this study was based on participants' self-reports of data that may be more subjective than objective. Secondly, the sample was drawn only from Chongqing, China and most of the adolescents were in early adolescence. Thus, the representation of older adolescents and the generalizability of the result are limited. Thirdly, this study was a cross-sectional survey, so causal conclusions cannot be drawn. Future studies will need a longitudinal research design to verify our findings. In addition, the relationship between NLEs and QoL is complex and future research should explore additional variables such as the mediating variables between NLEs and QoL.

## Conclusion

There was a significant negative correlation between NLEs and adolescents' QoL. Resilience and social support played an important mediating role between NLEs and QoL. Schools and society should strengthen health interventions that enhance adolescents' resilience and social support that in turn can improve their QoL.

## Data availability statement

The original contributions presented in the study are included in the article/supplementary material, further inquiries can be directed to the corresponding author/s.

## Ethics statement

The study was approved by the Ethical Committee of Chongqing Medical University. Written informed consent to participate in this study was provided by the participants' legal guardian/next of kin.

## Author contributions

HW and YT: conceptualization and writing-review and editing. HW, YT, and YM: methodology. HW, YT, YM, and JZ: formal analysis and investigation. YT: writing-original draft preparation. HW: funding acquisition. All authors read and approved the final manuscript.

## Funding

This study was supported by the Social Sciences Research Planning Fund Project from the Ministry of Education Humanities (17YJA840015).

## Conflict of interest

The authors declare that the research was conducted in the absence of any commercial or financial relationships that could be construed as a potential conflict of interest.

## Publisher's note

All claims expressed in this article are solely those of the authors and do not necessarily represent those of their affiliated organizations, or those of the publisher, the editors and the reviewers. Any product that may be evaluated in this article, or claim that may be made by its manufacturer, is not guaranteed or endorsed by the publisher.
